# Challenges in management of male breast adenomioepithelioma with malignant behavior: Case report

**DOI:** 10.1097/MD.0000000000017587

**Published:** 2019-10-25

**Authors:** Bogdan Gafton, Viorel Scripcariu, Iulian Prutianu, Teodora Alexa-Stratulat, Cristina Terinte, Andrei Nicolau, Diana Moisiuc, Iulian Radu

**Affiliations:** a “Grigore T. Popa” University of Medicine and Pharmacy Iaşi; b Regional Institute of Oncology Iaşi, Iaşi, Romania.

**Keywords:** breast cancer, man, malignant adenomyoepithelioma

## Abstract

**Rationale:**

Male adenomyoepithelioma of the breast with malignant features is a rare tumor with only one previous case reported in the literature over 25 years ago.

**Patient concerns:**

We report the case of a 63-year-old man admitted to our Oncology Institute with a painless tumor mass of 6 cm in the left breast with no additional regional lymph nodes. Ultrasound revealed a complex cystic tumor mass of 60 mm in the left breast, with both anechoic (cystic) and echogenic (solid) components, with ill-defined margin.

**Diagnoses:**

Extemporaneous assessment showed a solid (invasive) papillary intracystic carcinoma. Definitive pathology examination revealed the presence of a breast malignant adenomyoepithelioma.

**Interventions:**

Based on the extemporaneous assessment, wide tumor excision was performed. The tumor board decided to continue treatment with adjuvant anthracycline-based chemotherapy.

**Outcomes:**

After 6 years of follow-up, the patient is cancer-free. No chronic side effects were noted.

**Lessons:**

Because this pathology is extremely rare, no guidelines are available for its therapeutic approach. All decisions regarding patient management should be made by a multi-disciplinary team and can only be based on clinical experience and the few cases reported in female patients.

## 1 Introduction

Adenomyoepithelioma (AME) of the breast is a rare, salivary type, usually benign tumor most often diagnosed in women.^[1]^ AME of the breast was first describe by Hamperl in 1970 and further classified by Tavassoli in 1991.^[2,3]^

Breast AME is characterized by a biphasic proliferation of ephithelial and myoepithelial cells. Most breast AMEs are considered to be benign and those with more aggressive behavior tend to relapse locally. Malignant transformation is extremely rare and may involve one or both cellular components.^[4]^

Malignant adenomyoepithelioma of the breast is a malignant proliferation of the epithelial and myoepithelial cells, one or both components showing malignant features.^[5]^ The benign forms are cured with wide excision with negative margins, while malignant forms may require a multidisciplinary approach.^[6,7]^ Typically, AME appears as a single slowly growing nodule in the breast, followed by a rapid growing phase that leads to a clinical consultation.^[8]^ This is believed to be due to a malignant change one of the components, usually the epithelial one.^[5]^

Because this pathology is very rare, cases tend to be reported separately and no guidelines are available to help the clinician in deciding the optimal therapeutic approach. We report a case of breast AME in a male patient treated in the Regional Institute of Oncology Iasi, Romania. To our knowledge, it is the second case reported in literature, with the first one being presented over 25 years ago.^[9]^

## 2 Case presentation

**Figure 1:**
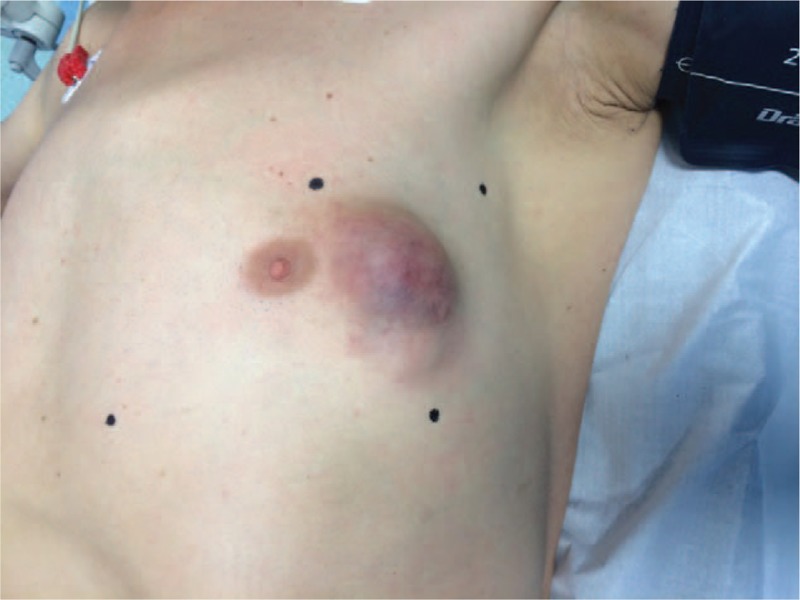
Clinical findings at diagnosis—left breast tumor, approximately 6 cm, adherent to the skin, but with no ulcerations.

A 63-year-old Caucasian man was admitted in 2012 to the Surgical Department of Iaşi Regional Institute of Oncology with a painless tumor mass of 6 cm in the left breast. The tumor had grown increasingly fast in the past 4 weeks and clinical examination identified inflammatory signs and skin invasion (Fig. 1). No lymph nodes were identified in the left axillae or in other regional stations. The patient had no relevant medical, family, or psychosocial history. The most important diagnostic concern was to determine the benign versus malignant nature of the tumor, which is why we first performed a breast ultrasound that revealed a complex cystic tumor mass of 60 mm in the left breast, with both anechoic (cystic) and echogenic (solid) components, with ill-defined margin. The anechoic component was dominant and there was a clear invasion of the skin.

After complete clinical staging, which showed no axillary lymph nodes and no systemic extension (cT3N0), the case was discussed in the multi-disciplinary tumor board (MDT). Because of the cystic character of the lesion, we decided to perform surgical resection and extemporaneous evaluation, rather than a tru-cut biopsy. The extemporaneous assessment showed a solid (invasive) papillary intracystic carcinoma which is a rare tumor composed of circumscribed large cellular nodules separated by bands of fibrosis.^[10]^ At this point, the surgeon decided to complete the resection and not to perform axillary lymph node dissection.

The histological report described 1 nodule with focal infiltrative margins, with a biphasic aspect due to the presence of 2 populations of cells. The lesion was composed of epithelial cells with oval-shaped nuclei, minute nucleolus, and eosinophilic cytoplasm. The epithelial cells were cuboidal or cylindric, and they were arranged in glandular-like structures, distorted, and compressed by the myoepithelial cells that were spindle or polygonal in shape with clear cytoplasm.

In some areas of the tumor, the myoepithelial cells were forming lobules separated by bands of fibrous tissue of variable size. In the myoephitelial component there was moderate nuclear atypia and 5/10HPF mitoses. The ki-67 index was positive in 30% of the cells in the most active areas.

**Figure 2:**
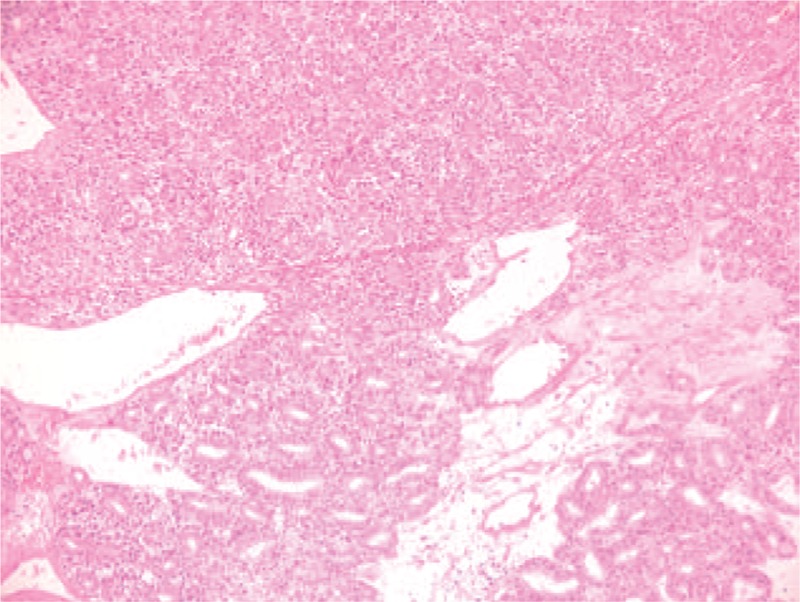
Microscopic image—solid tubular architecture with mioepitheial cells that surround the epithelial cells, HE coloration, 10×.

**Figure 3:**
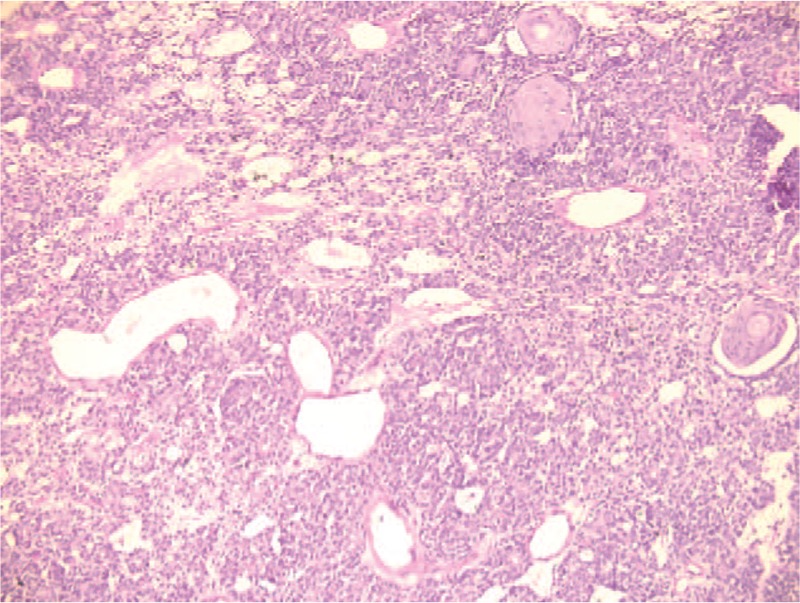
Microscopic image—mature squamous metaplasia, HE coloration, 10×.

The tumor also had myxochondroid areas (Fig. 2), squamous metaplasia with keratinisation (Fig. 3), and mucinous metaplasia. In some areas, we identified necrotic tissue. Also, satellite nodule of 1.2/1.5 cm with the same histological aspect as the primary tumor was seen.

**Figure 4:**
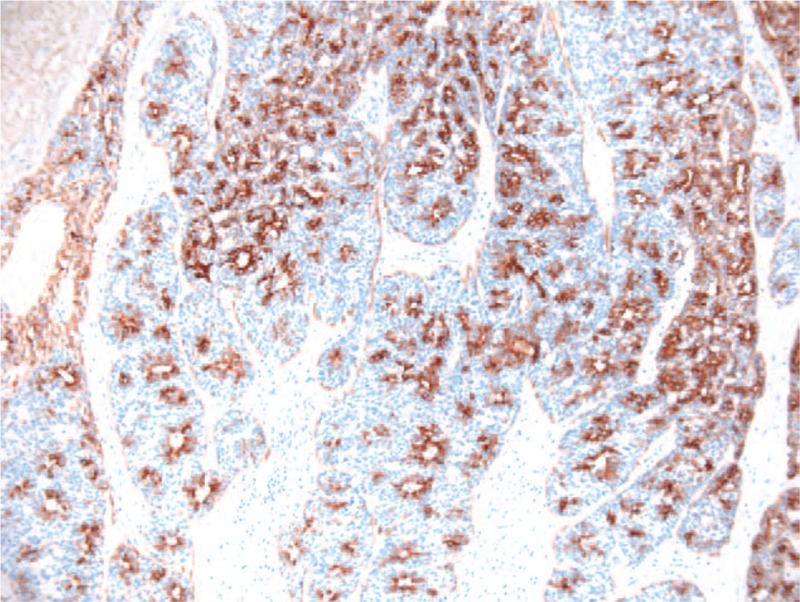
Microscopic image—cytokeratin (CK) 7 intense and diffuse positive in cells that tap the tubular elements, negative in mioepithelial cells, 10×.

**Figure 5:**
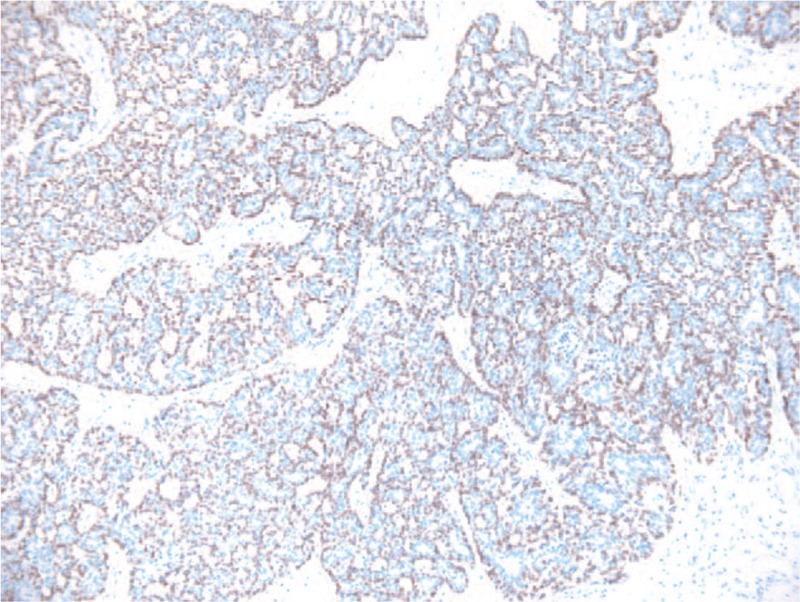
Microscopic image—p 63 stain positive for mioepithelial cells and negative for epithelial cells. HE coloration, 10×.

**Figure 6:**
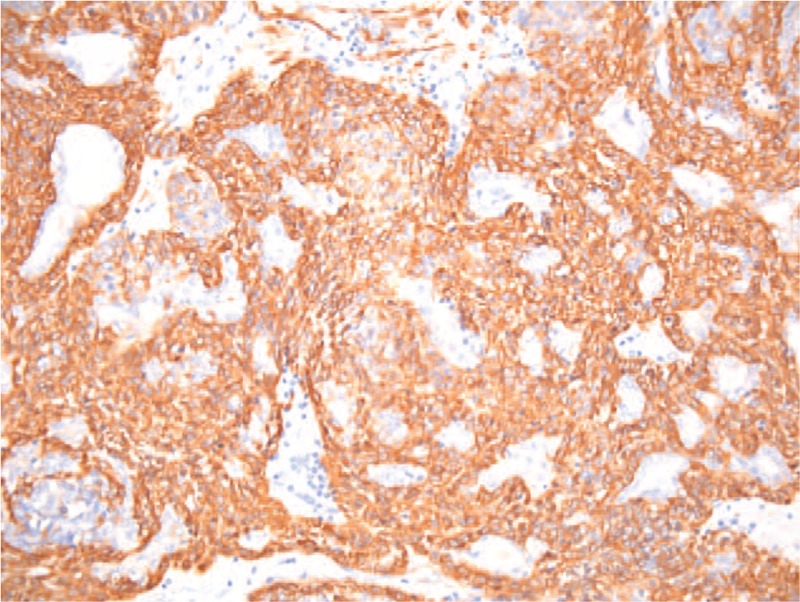
Microscopic image—smooth muscle actin (SMA) stain positive for mioepithelial cells and negative for epithelial cells. HE coloration, 20×.

The final pathology report after immunochemistry (Table 1, Figs. 4–6) suggested a malignant adenomyoepithelioma. Taking into account the size of the tumor (6 cm/5.5 cm), capsular invasion, satellite nodules, increased cellular proliferation in the myoepithelial component, moderate cellular pleomorphism, and the value of Ki-67 (30%) suggestive for malignant behavior, the pathologist concluded that the myoepithelial component had undergone a malignant transformation. Additionally, the tumor was estrogen receptor (RE) positive in 10% of the cells, progesterone receptor (PgR) negative, and Her2-neu negative (1+).

**Table 1:**
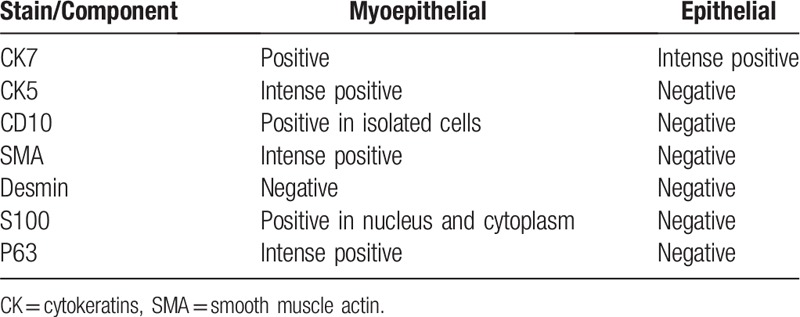
Immunochemistry stain.

The case was discussed again in our MDT. Since the behavior of such a tumor is difficult to predict, with possible recurrence and metastatic spreading, the board decided that the patient should receive 6 cycles of anthracycline-based adjuvant chemotherapy (doxorubicin 60 mg/m^2^ and cyclophosphamide 600 mg/m^2^, q 3 weeks). The treatment was well tolerated, with alopecia and grade I anemia as the main side effects. After 6 years of follow-up, the patient is free of disease.

## 3 Discussions

Malignant adenomyoepithelioma (MAME) of the breast is a malignant proliferation of the epithelial and myoepithelial cells, with one or both components showing malignant features; it arises from the myoepithelial and epithelial cells of the breast.^[5]^ The spreading pattern is most often hematogenous rather than lymphatic and metastatic disease usually occurs when primary tumors are >2 cm in size.^[11,12]^ Usually, the organs affected by metastases are lungs, brain, soft tissues, liver, bones, thyroid gland, and lymph nodes.^[8,13–15]^

In general, breast MAME appears as a palpable mass, with no lymph node involvement. On mammography, the tumor appears circumscribed, dense, and often has calcifications. On ultrasound, it appears as a well circumscribed homogenous solid nodule or as a cyst-solid lesion.^[5,16]^ In rare cases, the margins may be ill-defined.^[17]^ Features that suggest malignancy, such as adjacent ductectasia, may be present in some cases.^[18]^ If needed, high performance imaging tests such as MRI or CT scan can be recommended for better characterization of the lesion and for staging. The most common clinical differential diagnosis is with a benign breast tumor.

MAME is most often diagnosed after pathology analysis of the mastectomy piece. The fine-needle aspiration cytology (FNAC) may be useful in some cases, but may be very challenging because of the dispersed and mixed cells obtained by FNAC. FNAC may be misleading in most of the cases and false diagnosis like fibroadenoma or carcinoma may delay the right diagnosis.^[7,19]^

Microscopic analysis can suggest the presence of adenomyoepithelioma and the immunehistochemistry panel should include cytokeratins, S-100 protein, smooth muscle actin (SMA), calponin, p63 protein, Ki-67, EMA, Her, vimentin together with estrogen and progesteron receptor status.^[7]^ The markers should be evaluated in both epithelial and myoepithelial cells and are similar irrespective of origin site. The myoepithelial cells should express calponin, p63, SMA, CK5/6 and CK 14, p63, S-100, AE1, AE3 in various degrees. The estrogen and progesteron receptors should be negative or weakly positive while Her2 should be negative. The epithelial component is mostly positive for AE1, AE3, CK7, CK5/6, EMA, CAM5.2.^[20–22]^

From previously published cases, we know that this tumor most often occurs in the fifth and sixth decade^[13]^ and, similar to our case, initial presentation is a solitary breast mass identified by means of self-palpation.^[5]^ Due to its hematogenous spreading preference, the axillary lymph node dissection is not recommended, unless clinically palpable.^[8]^ The benign forms are cured with wide excision with negative margins, while malignant forms may require a multidisciplinary approach.^[6,7]^ In this case, no clinically palpable nodes were identified, which is why the surgical approach consisted in wide excision with negative margins.

Because most of the available knowledge in treating breast MAME is derived from the few published case reports or case series, the decision to include adjuvant chemotherapy in our patient's therapeutic management was based on the following consideration:

1. Malignant adenomyoepitelioma has potential for distant metastases (32% of cases),
2. A high incidence of metastatic disease apears in tumors >2 cm,
3. The incidence of metastases is higher in high-grade tumors (increased proliferation rate),
4. The malignant change was seen in the myoepithelial component,
5. Because the tumor had low expression of RE and is negative for PgR, we did not recomment adjuvant hormonal treatment. Some papers also suggest that hormonal treatment has no clear benefit.^[16]^

One additional difficulty in managing this case arose from the fact that our patient was man. To our knowledge, there has been only one case report involving breast adenomioepithelioma in a male patient that has been published over 25 years ago.^[9]^ In that case, the patient presented with a small tumor (1.3 × 0.9 cm) and was treated by means of surgical excision only. As such, this report is the first to describe the outcomes of surgery and chemotherapy for AME in a male patient. Due to the rarity of this condition, we do not know if this type of tumor is different in men when compared with women. However, based on data available from male breast cancer, we can speculate that MAME in men may be more aggressive^[23]^ and have a worst outcome^[24]^ when compared with MAME in women.

## 4 Conclusions

Malignant adenomioepithelioma in men are rare tumors. The management of this disease is not clearly defined and there are no guidelines for treatment. Due to the risk of metastatic spreading, we believe that malignant adenomyoepithelioma requires a multidisciplinary approach and a long follow-up.

## Acknowledgments

Patient has provided informed consent for publication of the case and the authors thank the patient for his permission to publish.

## Author contributions

**Conceptualization:** Bogdan Gafton, Viorel Scripcariu, Teodora Alexa-Stratulat, Andrei Nicolau, Iulian Radu.

**Data curation:** Viorel Scripcariu, Diana Moisiuc.

**Formal analysis:** Iulian Prutianu, Cristina Terinte, Iulian Radu.

**Investigation:** Bogdan Gafton, Viorel Scripcariu, Iulian Prutianu, Teodora Alexa-Stratulat, Cristina Terinte, Diana Moisiuc, Iulian Radu.

**Project administration:** Bogdan Gafton, Teodora Alexa-Stratulat, Cristina Terinte, Iulian Radu.

**Supervision:** Bogdan Gafton, Viorel Scripcariu, Teodora Alexa-Stratulat, Andrei Nicolau, Iulian Radu.

**Validation:** Viorel Scripcariu, Iulian Prutianu, Teodora Alexa-Stratulat, Andrei Nicolau, Diana Moisiuc.

**Visualization:** Viorel Scripcariu, Iulian Prutianu, Teodora Alexa-Stratulat.

**Writing – original draft:** Bogdan Gafton, Iulian Prutianu, Diana Moisiuc, Iulian Radu.

**Writing – review & editing:** Bogdan Gafton, Viorel Scripcariu, Teodora Alexa-Stratulat, Cristina Terinte, Andrei Nicolau, Iulian Radu.
